# Tumor Infiltrating Effector Memory Antigen-Specific CD8^+^ T Cells Predict Response to Immune Checkpoint Therapy

**DOI:** 10.3389/fimmu.2020.584423

**Published:** 2020-11-12

**Authors:** Nicola Principe, Joel Kidman, Siting Goh, Caitlin M. Tilsed, Scott A. Fisher, Vanessa S. Fear, Catherine A. Forbes, Rachael M. Zemek, Abha Chopra, Mark Watson, Ian M. Dick, Louis Boon, Robert A. Holt, Richard A. Lake, Anna K. Nowak, Willem Joost Lesterhuis, Alison M. McDonnell, Jonathan Chee

**Affiliations:** ^1^ National Centre for Asbestos Related Diseases, Institute for Respiratory Health, University of Western Australia, Nedlands, WA, Australia; ^2^ School of Biomedical Sciences, University of Western Australia, Crawley, WA, Australia; ^3^ Telethon Kids Institute, Perth, WA, Australia; ^4^ Institute of Immunology and Infectious Diseases, Murdoch University, Murdoch, WA, Australia; ^5^ Polpharma Biologics, Utrecht, Netherlands; ^6^ BC Cancer Agency, Vancouver, BC, Canada; ^7^ School of Medicine, University of Western Australia, Crawley, WA, Australia

**Keywords:** immune checkpoint therapy, tumor-specific T cells, TCR repertoire, cytotoxic T lymphocytes, effector memory

## Abstract

Immune checkpoint therapy (ICT) results in durable responses in individuals with some cancers, but not all patients respond to treatment. ICT improves CD8^+^ cytotoxic T lymphocyte (CTL) function, but changes in tumor antigen-specific CTLs post-ICT that correlate with successful responses have not been well characterized. Here, we studied murine tumor models with dichotomous responses to ICT. We tracked tumor antigen-specific CTL frequencies and phenotype before and after ICT in responding and non-responding animals. Tumor antigen-specific CTLs increased within tumor and draining lymph nodes after ICT, and exhibited an effector memory-like phenotype, expressing IL-7R (CD127), KLRG1, T-bet, and granzyme B. Responding tumors exhibited higher infiltration of effector memory tumor antigen-specific CTLs, but lower frequencies of regulatory T cells compared to non-responders. Tumor antigen-specific CTLs persisted in responding animals and formed memory responses against tumor antigens. Our results suggest that increased effector memory tumor antigen-specific CTLs, in the presence of reduced immunosuppression within tumors is part of a successful ICT response. Temporal and nuanced analysis of T cell subsets provides a potential new source of immune based biomarkers for response to ICT.

## Introduction

Cancer immunotherapies that block inhibitory checkpoint receptors on T cells, such as cytotoxic T-lymphocyte associated protein 4 (CTLA-4) and programmed death receptor 1 (PD-1), have resulted in remarkable, long-term tumor control in a subset of patients (1–3). However, the majority of ICT-treated patients do not benefit. ICT is very expensive and causes immune-related toxicities. Accordingly, there is an urgent need for sensitive and specific biomarkers of response. Current biomarkers include the expression of checkpoint inhibitory ligands such as PD-L1 (4), tumor mutation burden (5), gene expression profiles of the tumor microenvironment (6), and the extent of tumor infiltrating immune cells (7). Each biomarker has its own strengths and limitations, but there is currently no accurate predictor of responsiveness to ICT across multiple cancers. Developing novel, complementary biomarkers associated with successful response to ICT will guide clinical decisions and help understand the underlying immune mechanisms of a successful anti-tumor immune response (8).

Analysis of CD8^+^ cytotoxic T lymphocytes (CTL) could offer a biomarker of response to ICT. Inhibitory checkpoint signaling that occurs through the PD-1/PD-L1 pathway suppresses activated CTLs within the tumor microenvironment, preventing tumor cell killing. ICT drives dynamic changes in CTL frequency (7, 9), phenotype (10–12), proliferation (13, 14), and cytotoxic function (6, 15). T cell receptor (TCR) sequencing studies further suggest that ICT causes clonal proliferation of CTLs within the tumor (7, 16, 17) and the periphery (16, 18, 19). As antigen-specificity is crucial for a successful anti-tumor response, we reasoned that dynamic changes in tumor antigen-specific CTLs could inform ICT responses. Indeed, ICT can increase tumor antigen-specific CTLs (20, 21), but there are limited studies on how ICT-driven phenotypic changes in tumor antigen-specific CTLs correlate with ICT outcomes.

The ability to study how tumor antigen-specific CTLs contribute to ICT outcomes in clinical studies is limited because of variability in host genetics (which includes TCR repertoire), clinical history, tumor mutations, and antigen expression. Furthermore, serial tumor biopsies are often not feasible, making it difficult to assess dynamic changes within the tumor microenvironment. Murine models are useful in this context because variation can be controlled. We used a model in which inbred mice bearing tumors derived from monoclonal cancer cell lines respond dichotomously to anti-CTLA-4 and anti-PD-L1 ICT, with some mice experiencing complete tumor regression within days, and the others not responding to therapy (22). We previously defined a pre-treatment ICT responsive gene signature in the tumor microenvironment using this model (23). In the present study, we characterized CTLs specific for a model tumor antigen using this established model, correlating dynamic changes in T cell frequencies, phenotype, and clonality to ICT outcomes. We identified effector memory CTL phenotypes that can be further tested as immune biomarkers of ICT response.

## Materials and Methods

### Mice

BALB/c.Arc and BALB/c.AusBP mice were bred and maintained at the Animal Resource Centre (ARC; Murdoch, WA, Australia) or Harry Perkins Institute of Medical Research (Nedlands, WA, Australia). Clone 4 (CL4xThy1.1) TCR transgenic mice express a TCR that recognizes a MHC class I-restricted influenza A/PR/8 hemagglutinin (HA_533-541_) epitope (24). As >97% of CD8^+^Thy1.1^+^ T cells expressed the transgenic TCR, Thy1.1 was used as a surrogate marker to track HA-specific CD8^+^ T cells. CL4xThy1.1 mice were kindly provided by Prof Linda Sherman (The Scripps Research Institute, La Jolla, CA) and bred at the Animal Resource Centre (ARC). All mice used in these studies were between 8 and 10 weeks of age and were maintained under standard specific pathogen free housing conditions at the Harry Perkins Bioresources Facility (Nedlands, WA, Australia). All animal experiments were carried out in accordance with the Harry Perkins Institute of Medical Research Animal Ethics guidelines and protocols (AE140).

### Transfer of TCR Transgenic Splenocytes

Spleens from CL4xThy1.1 mice were manually dissociated through 40 µm strainers with phosphate-buffered saline (PBS) supplemented with 2% Newborn Calf Serum (NCS; Life Technologies). Red blood cells were lysed with Pharm Lyse (BD Biosciences) and splenocytes were washed twice with PBS. Mice were intravenously injected with 1 x 10^6^ splenocytes suspended in 100 µl of PBS 24 h prior to tumor inoculation.

### Cell Lines

The murine malignant mesothelioma cell line AB1 (25) was transfected with influenza hemagglutinin (HA) from the Mt Sinai strain of PR8/24/H1N1 influenza virus to generate the AB1-HA cell line (26) (CBA, Cat# CBA-1374, RRID: CVCL_G361). AB1 and renal cell carcinoma (RENCA) cell lines were used for re-challenge and ex-vivo co-culture experiments. RENCA was obtained from ATCC (ATCC, Cat# CRL-2947, RRID: CVCL_2174) and AB1 was obtained from Cell Bank Australia (CBA, Cat# CBA-0144, RRID: CVCL_4403). Cell lines were maintained in R10; RPMI 1640 (Invitrogen) supplemented with 20 mM HEPES (Gibco), 0.05 mM 2-mercaptoethanol (Sigma Aldrich), 100 units/ml benzylpenicillin (CSL), 50 µg/ml gentamicin (David Bull Labs), 10% NCS (Life Technologies) and 50 mg/ml of geneticin for AB1-HA only (G418; Life Technologies). Cells were grown to 80% confluence before passage and passaged three to five times before inoculation.

### Tumor Cell Inoculation

Cells were harvested when they reached 80% confluence. Mice were inoculated subcutaneously (s.c.) into the shaved, right-hand flank (for single inoculations) or both left- and right-hand flanks (for dual-tumor inoculations) with 5 x 10^5^ tumor cells suspended in 100 µl of PBS using one 26-gauge needle per injection (22). Length and width tumor measurements were monitored using calipers to calculate tumor area (mm^2^).

### Immune Checkpoint Therapy

Immune checkpoint antibodies anti-CTLA4 (clone 9H10) and anti-PD-L1 (clone MIH5) were prepared and purified by Polpharma Biologics (Urecht, Netherlands) as previously described (23). Mice received an intraperitoneal injection (i.p.) of 100 µg of anti-CTLA4 and 100 µg of anti-PD-L1. 100 µg of anti-PD-L1 was subsequently administered 2 and 4 days after the initial dose, as previously optimized (22). Control mice received PBS at the equivalent volume, as previous work found no difference between control immunoglobulin G and PBS (27). Mice were randomized before treatment. The first dose of ICT was administered 7 to 10 days after tumor inoculation, when tumors were between 9 and 20 mm^2^ in size. Mice were defined as responders when their tumor completely regressed and remained tumor-free for at least 4 weeks after treatment. Mice were designated as non-responders if their tumors grew to 100 mm^2^ within 4 weeks after the start of therapy, similar to PBS controls. Mice that had a delay in tumor growth or partial regression were designated as intermediate responders and excluded from the analysis. We only used experiments in which mice displayed a dichotomous response, where there had to be at least one non-responder and one responder in each cage (22, 23).

### Surgical Excision of Lymph Nodes and Complete Tumor Debulking

Complete tumor debulking and lymphadenectomy of the right-hand flank was performed either on the day of treatment (prior to ICT administration; day 0) or 7 days post-therapy (day 7), as previously described (22). Briefly, mice were dosed with 0.1 mg/kg of buprenorphine in 100 µl, 30 min before anaesthesia with isoflurane (4% in 100% oxygen at a flow rate of 2 liters/min). Whole tumors and draining inguinal lymph nodes (DLN) were surgically excised. Surgical wounds were closed using Reflex wound clips (CellPoint Scientific). Mice received subsequent doses of 0.1 mg/kg of buprenorphine in 100 µl 6 and 24 h after surgery for pain relief.

### Preparation of Single Cell Suspensions

DLNs were manually dissociated through 40 µm strainers with PBS + 2% NCS. Tumors were dissected into smaller pieces with a scalpel blade and subjected to digestion with 1.5 mg/ml type IV collagenase (Worthington Biochemical) and 0.1 mg/ml type I DNAse (Sigma Aldrich) in PBS + 2% NCS for 45 min at 37°C on a Microtitre Plate Shaker Incubator (Thomas Scientific) (28). Tumors were washed twice in PBS + 2% NCS following digestion. Cell counts were performed using a hemocytometer with trypan blue exclusion.

### T Cell: Tumor Co-Culture

Spleens from ICT responders were harvested 14 days post re-challenge with cell line AB1-HA. Splenocytes were seeded at a density of 1 x 10^6^ cells/well in a 96-well plate and stimulated with cell lines; AB1-HA, AB1 or RENCA, or the HA peptide at a 10:1 effector:target ratio for 20 h at 37°C. Brefeldin A (Biolegend) was added into each well for the last 4 h of the culture. Cells were washed twice with PBS + 2% NCS before antibody staining.

### Flow Cytometry

Four flow cytometry panels outlined in **Table S1** were performed. Samples were stained with Fixable Viability Dye (FVD) eFluor™ 506 (eBioscience) or Zombie UV™ (BioLegend) to exclude dead cells. Cells were incubated with Zombie UV™ suspended in PBS in the dark for 30 min at room temperature (RT) prior to staining with surface antibodies. Antibodies for surface staining (including FVD eFluor™ 506) were suspended in PBS + 2% NCS and incubated on cells for 30 min at 4°C. PBS + 2% NCS was used to wash cells between incubations. Samples were then fixed and permeabilised for 10 min at 4°C using the Foxp3/Transcription Factor Staining Buffer Set (eBioscience). Cells were washed with Permeabilization Buffer (eBioscience), subjected to intracellular staining and left overnight at 4°C. Single stain and fluorescence minus-one (FMO) controls were also used. Data were acquired using a BD LSRFortessa™ SORP or BD FACSCanto II™ (BD Biosciences) with 50,000 live events collected per sample where possible. All flow cytometry analyses were completed using FlowJo™ Software version 10 (BD Biosciences). Summary of antibodies concentrations and gating strategies is outlined (**Table S1**, **Figure S8**).

### Fluorescence Activated Cell Sorting

Tumors were stained for fluorescence activated sorting using the BD FACSMelody™ cell sorter (BD Biosciences). All samples were stained with antibodies outlined in **Table S1** for 30 min at 4°C to sort for CD8^+^ T cells for TCRβ sequencing or CD8^+^Thy1.1^+^ T cells for RNA sequencing. Sorted cells were collected in 500 µl of RNAprotect cell reagent (QIAGEN) and stored at −20°C. Sorts were run on greater than 85% efficiency. Sorting gates are described in **Figure S9**.

### Bulk TCRβ Sequencing

TCRβ libraries were made using a 5’Rapid Amplification of cDNA Ends (5’RACE) technology optimised from R. Holt and colleagues (29). RNA was extracted from cell sorted samples using the RNeasy Plus Micro Kit (QIAGEN). Total RNA was transcribed to cDNA using a TCRβ constant region primer (Integrated DNA Technologies) and a modified SMARTerIIA primer (Integrated DNA Technologies), adding unique molecular identifiers (UMI) to individual TCRβ cDNA sequences for unbiased PCR amplification. The TCRβ locus was amplified by nested PCR with another TCRβ constant region primer (Integrated DNA Technologies), and a universal primer to SMARTerIIA, with the final PCR adding sequencing adaptors and barcodes to the TCRβ libraries. PCR products were purified using AMPure XP AgenCourt Beads (Beckman Coulter).

Paired-end (2 × 300 bp) high-throughput sequencing was performed using the Illumina MiSeq platform (Illumina, RRID:SCR_016379). Data processing, aggregation of UMIs and alignment of CDR3 sequences to the IMGT/V-QUEST reference genome (30) were performed using repertoire analysis software based on MIGEC (31) (RRID:SCR_016337) and MiXCR (32) (RRID:SCR_018725) pipelines. Only sequences with UMIs were aligned. In-house analysis tools used were provided by AC and MW (Institute for Immunology and Infectious Diseases, Murdoch, Australia).

### TCRβ Repertoire Analysis

TCRβ libraries were analyzed using functions in R (R Project for Statistical Computing, RRID: SCR_001905, v3.6.0). A TCRβ clone was defined by the CDR3 amino acid sequence. Clones that were less than 8 or greater than 20 amino acids in length, included a stop codon or a frameshift were defined as non-functional and were excluded from analysis. To measure TCRβ repertoire diversity, Renyi entropy was used given by $H_{\alpha }(X)=\frac{1}{1-\alpha }\log(\Sigma_{i=1}^{n}\;p_{i}^{a})$ where α is a scale of values, ranging from 0 to infinity. The closer α gets to infinity, the more weight is given to more abundant TCRs. α = 0 corresponds to ‘richness’, the number of unique TCRβ sequences (TCRβ clones). α = 1 corresponds to Shannon’s entropy. α = 2 corresponds to Simpson’s diversity (33). Shannon’s entropy was also calculated by; $H=1-\frac{\left(-\Sigma_{i=1}^{N}\;p_{i}lnp_{i}\right)}{n}$ where p_i_ is the proportion of sequence *i* relative to the total *N *sequences (34). This index ranges from 0 to 1; 0 being an entirely monoclonal sample, and 1 meaning each unique TCRβ clone only occurs once. Networks of the most abundant TCRβ clones were constructed using the *ggraph* extension (v2.0.2) of *ggplot2* package in R (RRID:SCR_014601, v3.2.1). Each node in the network represents a unique CDR3 TCRβ sequence. Each edge is defined as a single amino acid difference (levenshtein distance of 1) between the CDR3 TCRβ sequences (35). TCRβ CDR3 sequence for the CL4 clone (CASGETGTNERLFF) was determined by bulk TCRβ sequencing of sorted CD8^+^ splenocytes from CL4xThy1.1 mice.

### Bulk RNA Sequencing

RNA was extracted from CD8^+^Thy1.1^+^ cell sorted samples using the RNeasy Plus Micro Kit (QIAGEN). RNA quality was confirmed on the Bioanalyzer (Agilent Technologies). Library preparation and sequencing on the Novaseq 550 (75 base pair, paired-end, Illumina), quality assessment using FastQC and alignment to the GRCm38/mm10 mouse reference genome were performed by the Institute for Immunology and Infectious Diseases (Murdoch, Australia). The Broad Institute Inc. Gene Set Enrichment Analysis (GSEA) Software (RRID:SCR_003199, v4.0.2) was used to analyze 50 MSigDB hallmark gene sets on normalized gene expression data (36). Gene sets enriched at a nominal P < 0.05 and FDR < 0.25 were considered significant.

### Statistical Analysis

Data are presented as mean ± SD. Mann-Whitney *U* tests were used for comparisons between the means of two variables. Ordinary Two-way ANOVA with Sidak’s multi-comparisons was used to compare the interaction between two variables. Correlation was analyzed using Pearson correlation tests. Kaplan-Meier method was used for survival analysis with log-rank test (Mantel-Cox) to analyze significance. All statistics was performed using GraphPad Prism Software (Graph Pad Software Inc., RRD:SCR_002798, v8). Results were significant when p < 0.05 (*p < 0.05, **p < 0.01, ***p < 0.001, ****p < 0.0001).

## Results

### The Frequency of Tumor Antigen-Specific CD8^+^ TILs Is Highly Variable Irrespective of Response Phenotype

**Figure 1:**
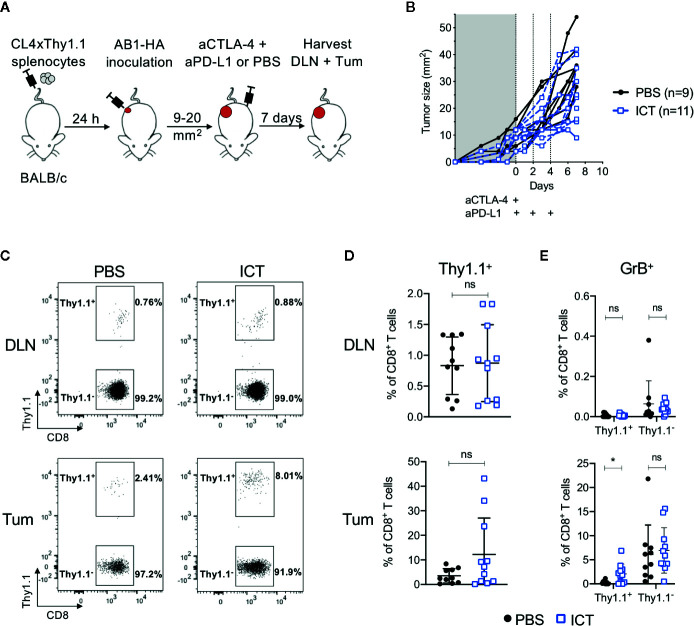
ICT increases tumor infiltrating cytotoxic tumor antigen-specific CD8^+^ T cells. **(A)** Experimental timeline. CL4xThy1.1 splenocytes were adoptively transferred into BALB/c mice one day prior to AB1-HA tumor inoculation. Mice were treated with ICT (aCTLA-4 and aPD-L1) or PBS when tumors reached 9 to 20 mm^2^ in size. Tumors (Tum) and corresponding draining lymph nodes (DLN) were harvested 7 days post-treatment. **(B)** Tumor growth curves of mice treated with PBS (black) or ICT (blue). Each line represents an individual animal. Dotted lines indicate days of treatment. **(C)** Representative FACS plots, and **(D)** dot plots representing frequencies of CD8^+^Thy1.1^+^ (HA-specific) T cells in DLN and Tum of both treatment groups. **(E)** Frequency of granzyme B (GrB) expressing CD8^+^Thy1.1^+^ or Thy1.1^−^ T cells. Data in dot plots represented as mean ± SD. Mann-Whitney *U* tests were used to compare groups; *P ≤ 0.05. Data represents two independent experiments.

To track how ICT changes the frequency and phenotype of tumor antigen-specific CD8^+^ T cells, we transferred T cells specific for a MHC-I restricted HA_533-541_ antigen from CL4xThy1.1 mice (24) into BALB/c recipient mice prior to inoculation of a HA expressing tumor cell line (26). Mice were treated with anti-CTLA-4 and anti-PD-L1, and HA-specific (CD8^+^Thy1.1^+^) T cells in tumors (Tum) and draining lymph nodes (DLN) post therapy were analyzed (**Figures 1A, B**).

Overall, there was no significant difference in frequency of CD8^+^Thy1.1^+^ and CD8^+^Thy1.1^−^ T cells between ICT treated and control groups. However, CD8^+^Thy1.1^+^ T cells tended to increase in the tumors of ICT treated mice (**Figures 1C, D**). Recipient and donor CD8^+^ T cells in DLNs expressed minimal granzyme B (GrB), regardless of treatment (**Figure 1E**). The number of CD8^+^Thy1.1^+^GrB^+^ TILs were significantly higher in the ICT treated group (P = 0.013), but this difference was not found in endogenous CD8^+^ T cell populations, suggesting that our ICT regime increased the cytotoxic function of HA-specific CTLs.

Interestingly, the number of HA-specific CTLs varied between ICT treated animals, making up greater than 20% of CD8^+^ TIL populations in some tumors, and less than 5% in others. This suggests that although all tumors expressed HA (26), the frequencies of HA-specific CTLs did not increase in all animals after ICT.

### A Unique Murine Bilateral Tumor Model to Track Tumor Antigen-Specific CTLs in ICT Outcomes

**Figure 2:**
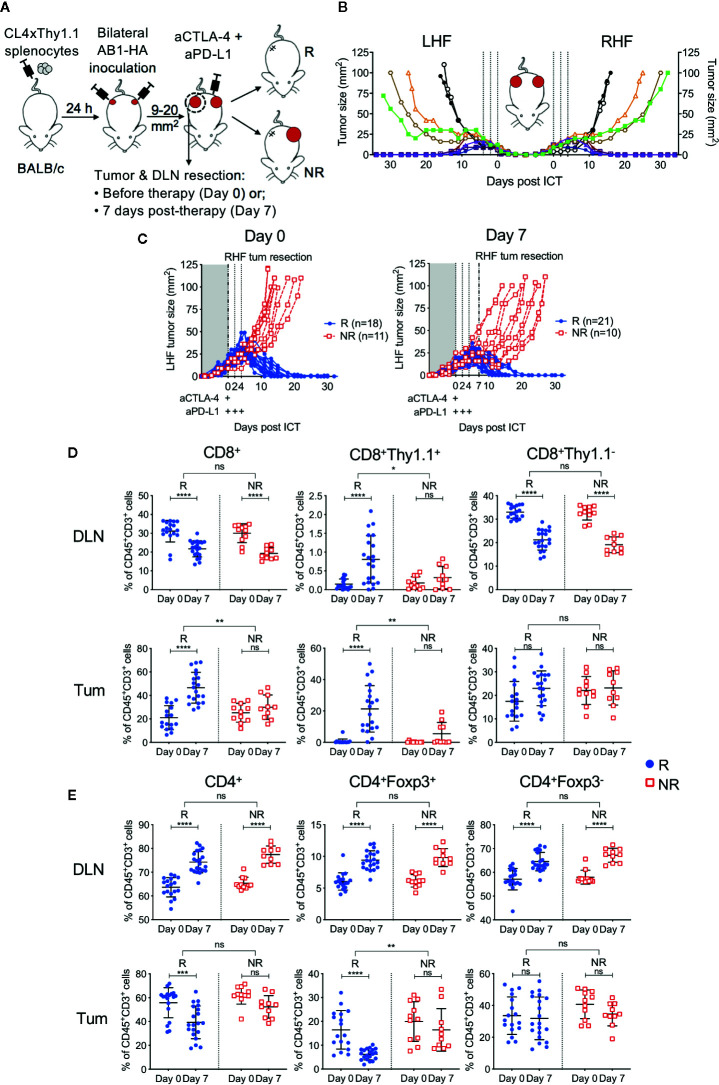
Tumor antigen-specific CD8^+^ T cells increase in ICT responding DLN and tumors. **(A)** Experimental timeline. CL4xThy1.1 splenocytes were adoptively transferred into BALB/c mice one day prior to bilateral AB1-HA tumor inoculation. Right-hand flank (RHF) tumor (Tum) and draining lymph node (DLN) were surgically resected either pre- (day 0) or post-ICT (day 7). Left-hand flank (LHF) tumor was followed for ICT response. **(B)** Growth curves representing symmetrical growth and regression of bilateral AB1-HA tumors treated with ICT (n = 8; color-coded per mouse) or PBS (n = 2; black), without surgery. Dotted lines indicate days of treatment. **(C)** Growth curves of LHF tumors for mice that had their RHF tumors and DLNs resected at day 0 (left) or day 7 (right). Mice were characterized as responders (R; blue) or non-responders (NR; red). Dotted lines indicate days of treatment. Pre (Day 0) and post (day 7) treatment frequencies of total CD8^+^, CD8^+^Thy1.1^+^ and CD8^+^Thy1.1^−^ **(D);** total CD4^+^, CD4^+^Foxp3^+^ and CD4^+^Foxp3^-^ T cells **(E)** in resected DLNs (top) and tumors (bottom) of responding and non-responders. Data represented as mean ± SD, summary of five independent experiments. Two-way ANOVAs were used to compare the magnitude of difference between responders and non-responders, with Tukey’s multiple-comparisons to compare pre- and post-treatment frequencies within each group; *P ≤ 0.05, **P ≤ 0.01, ***P ≤ 0.001, ****P ≤ 0.0001.

To have more certainty on the presence or absence of a correlation between the frequency of HA-specific CTLs and outcome to ICT, we utilized our established bilateral tumor model where inbred, age-matched mice harboring monoclonal tumors display dichotomous responses to ICT (22) (**Figure 2A**). Importantly, the addition of CL4xThy1.1 splenocytes did not alter ICT response rate or symmetry in the bilateral model (**Figures 2B**, **S1**). Symmetry in tumor growth and regression upon ICT allowed us to surgically remove one tumor and its corresponding DLN to track HA-specific CTLs, while tracking how this tumor would have responded to ICT, by monitoring the contralateral tumor.

Tumors and their corresponding DLNs were resected for analysis either prior to ICT administration (day 0) or 7 days after (day 7; **Figure 2A**). At day 0, excised tumors were indistinguishable by size, total cell count, and proportions of CD45^+^ cells regardless of subsequent response outcomes (**Figure S2A**). At day 7, non-responding tumors were greater in size than responding tumors (P = 0.003), however total cell counts and proportions of CD45^+^ cells were similar between groups (**Figure S2A**). Dichotomous responses to ICT were observed, with tumors reaching 100 mm^2^ in non-responders (NR; red), or completely regressing to 0 mm^2^ in responders (R; blue) by 20 days post-treatment (**Figure 2C**).

### ICT Responders Have More Tumor Antigen-Specific CTLs in Tumors and Draining Lymph Nodes, and Reduced Intra-Tumoral T_regs_ Compared to Non-Responders

In responding mice, the number of CD8^+^ TILs were significantly higher after treatment (**Figure 2D**). In contrast, non-responding animals had a similar amount of CD8^+^ TILs pre- and post-treatment (**Figure 2D**; R vs NR; DLN: P > 0.05; Tum: P = 0.0011). The number of HA-specific CD8^+^Thy1.1^+^ T cells in DLNs and tumors were significantly higher after treatment in responding, but not in non-responding mice (**Figure 2D**; R vs NR; DLN: P = 0.02; Tum: P = 0.004). Endogenous CD8^+^Thy1.1^−^ T cells significantly decreased after treatment in DLNs, but remained similar in tumors in both responders and non-responders (**Figure 2D**).

The number of total CD4^+^ T cells, T_regs_ (CD4^+^Foxp3^+^) and helper T cells (CD4^+^Foxp3^-^) in the DLN increased after treatment in both responding and non-responding animals (**Figure 2E**). Intra-tumoral CD4^+ ^T cell frequencies decreased post-ICT in responders, which was largely attributed to a reduction in T_regs_. This reduction was not observed in non-responders (**Figure 2E**, R vs NR; DLN: P > 0.05; Tum: P = 0.002). Intra-tumoral CD4^+^Foxp3^+^ frequencies inversely correlated to the proportion of CD8^+^Thy1.1^+^ T cells after treatment (**Figure S3**; r = −0.393; P = 0.03). Taken together, our data suggests that a post‑treatment increase in HA-specific CD8^+^ T cells in DLN and tumors, accompanied by a reduction in intra-tumoral T_regs_ is associated with ICT response.

### Responding Tumors Have a More Clonal TCRβ Repertoire Compared to Non-Responders, but Each Animal Uses a Private CD8^+^ TIL Repertoire

**Figure 3:**
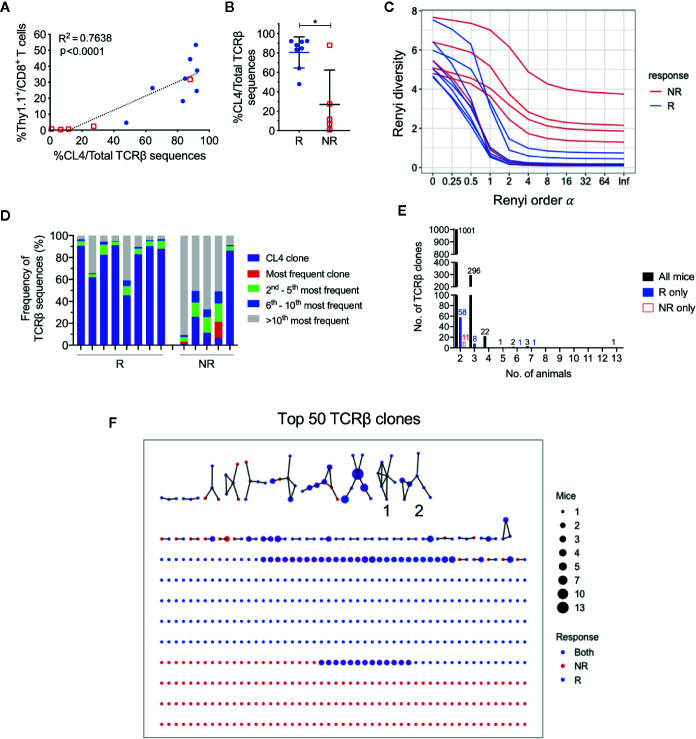
CL4 transgenic TCRβ clone dominated post-treatment CD8^+^ TIL TCRβ repertoire in responding animals. **(A)** Linear regression analysis between the CL4 TCRβ clone frequency in TCRβ sequencing and the frequency of CD8^+^Thy1.1^+^ T cells analyzed in flow cytometry. **(B)** Dot plot representing the CL4 TCRβ clone frequencies in responders (R; blue) and non-responders (NR; red). **(C)** Graph of Renyi diversity profiles for each TCRβ repertoire. The scale of Renyi order α corresponds to calculated diversity metrics. α = 0 indicates the richness of the repertoire (number of unique TCRβ clones). Shannon’s diversity index corresponds to α = 1. Each line represents the Renyi entropy of one animal, and a steeper gradient between α = 0 and 1 represents a less diverse repertoire. **(D)** Bar graph displaying proportions of the 10 most frequent TCRβ clones in responders and non-responders. Each bar represents the TCRβ repertoire of one animal. CL4 clone (purple) is the most frequent clone in 11/13 animals. **(E)** Bar graphs representing the number of shared TCRβ clones between 2 or more animals. Shared clones are separated into overlap within only responders (blue), only non-responders (red), or all mice regardless of outcome (black). **(F)** Network analysis of the top 50 most abundant TCRβ clones for each animal. Each node represents a unique CDR3 TCRβ sequence (TCRβ clone) and each edge defines a single amino acid difference (levenshtein distance of 1). Size of each node represents the number of mice that have the TCRβ clone in their repertoire and nodes are colored by presence of TCRβ clone in only responders (blue), only non-responders (red) or both groups (purple). Data is shown as mean ± SD where appropriate; R (n = 8) and NR (n = 5) were sampled from three independent experiments; Mann-Whitney *U* tests; *P ≤ 0.05.

As responding animals had increased frequencies of CTLs specific against one tumor antigen, we next examined if there was oligoclonal expansion of other CTL specificities in the endogenuous population. To characterize TCR repertoires of post-treatment CD8^+^ TILs, we performed bulk TCRβ sequencing on sorted CD8^+^ TIL populations. The total number of sorted cells significantly correlated with the total number of TCRβ sequences returned (**Figure S4A**; r = 0.89, P < 0.0001), and the total number of sorted cells, unique and total TCRβ sequences were similar between responders and non-responders (**Figure S4B**). The frequency of HA-specific CD8^+^ T cells identified by flow cytometry also significantly correlated with the number of CL4 TCRβ CDR3 sequences (CASGETGTNERLFF) in matched samples (**Figure 3A**; r = 0.87, P < 0.0001), highlighting that the most abundant TCR clones were being captured by our TCRseq assay.

Consistent with our previous experiments, the proportion of post-treatment CL4 TCRβ sequences was significantly greater in responders (80.5 ± 16.1%) than non-responders (26.9 ± 35.5%) (**Figure 3B**; P = 0.03). We estimated the diversity of TCRβ repertoires by their Renyi entropies (**Figure 3C**), and found that responders had significantly less diverse TCRβ repertoires than non-responders (**Figure S4C, D**; P = 0.019), suggesting that expansion of the CL4 clone correlated with response. The CL4 clone was the most frequent TCRβ clone for all responding animals (47.9–92.3%) and the majority of non‑responding animals (11.6–87.9%). The subsequent most abundant TCR clonotypes (2^nd^ to 10^th^) varied between animals, making up 3.72% to 27.6% of each repertoire (**Figure 3D**), suggesting that expansion within responding tumors was mostly monoclonal.

As all mice harbored a tumor that expressed a common antigen (HA), we examined the overlap between tumor-infiltrating CD8^+^ TCRβ clones between animals. Excluding the transferred CL4 clone, there was minimal sharing of TCRβ clones between all animals, regardless of response. The majority of overlapping clones were shared between two and three animals (**Figure 3E**). We next represented the top 50 most abundant TCRβ clones from each animal in a network based on their TCRβ CDR3 amino acid sequence similarities. 57 out of 560 TCRβ clones formed networks, but the majority of TCRβ clones were not related to any other clone, failing to form any networks (**Figure 3F**). Most TCRβ CDR3 sequences were randomly distributed throughout the networks regardless of response, with the exception of two groups of highly similar TCRβ CDR3 sequences that were exclusively found in responding tumors (Group 1, 2, **Figure 3F**). However, each clone from these groups was present in only one to three responding mice and comprised less than 0.6% of the TCRβ repertoire.

These data suggest that apart from the clone we introduced, each animal had a private and highly diverse tumor-infiltrating CD8^+^ TCRβ repertoire. Importantly, responders had a less diverse CD8^+^ TCR repertoire compared to non-responders, but this was largely attributed to the expansion of HA-specificity in this model.

### ICT Responders Have Increased Post-Treatment Tumor Antigen-Specific Effector Memory CTLs Compared to Non-Responders

We next investigated CD8^+^ T cell phenotype in ICT responders and non-responders. Endogenous (Thy1.1^−^) and transferred (Thy1.1^+^) CD8^+^ T cells were analyzed for expression of differentiation and memory-associated markers. We focused on post-treatment (day 7) because pre-treatment DLN and tumors contained <1% of CD8^+^Thy1.1^+^ T cells (**Figure 2D**), making it difficult to accurately analyze their phenotype.

**Figure 4:**
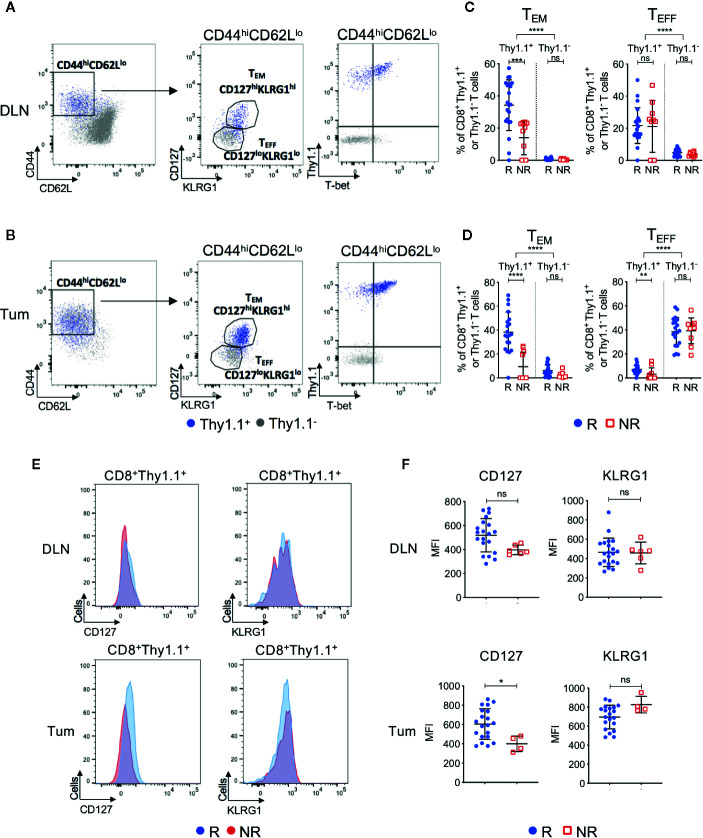
Tumor infiltrating tumor antigen-specific CD8^+^ T cells acquire an effector memory phenotype in ICT responding animals. Representative FACs plots comparing CD8^+^Thy1.1^+^ (blue) and CD8^+^Thy1.1^−^ (gray) T cell phenotype in post-treatment **(A)** DLNs and **(B)** tumors. Cells were analyzed for CD44, CD62L, CD127, KLRG1 and T-bet expression. Gates on the FACS plot represent effector memory (T_EM_; CD44^hi^CD62L^lo^CD127^hi^KLRG1^hi^) and effector (T_EFF_; CD44^hi^CD62L^lo^CD127^lo^KLRG1^lo^) T cell subsets. Graphs representing frequencies of tumor antigen-specific (CD8^+^Thy1.1^+^) and endogenous (CD8^+^Thy1.1^−^) T cells that exhibit T_EM_ or T_EFF_ phenotypes in **(C)** DLNs and **(D)** tumors, grouped by response/non-response to ICT. **(E)** Representative histograms comparing CD127 and KLRG1 expression on activated (CD44^hi^CD62L^lo^) CD8^+^Thy1.1^+^ T cells between responding and non-responding DLNs (top) and tumors (bottom). **(F)** Median fluorescence intensity (MFI) expression of CD127 and KLRG1 on CD8^+^Thy1.1^+^ T cells in DLNs (top) and tumors (bottom) represented as dot plots. Data shown as mean ± SD. Mann-Whitney *U* tests were used to compare between both responders and non-responders, and between Thy1.1^+^ and Thy1.1^−^ T cells for each T cell phenotype; *P ≤ 0.05, **P ≤ 0.01, ***P ≤ 0.001, ****P ≤ 0.0001.

Post-treatment CD8^+^Thy1.1^+^ T cells were activated(CD44^hi^CD62L^lo^) and upregulated memory and differentiation markers IL-7Rα (CD127), killer cell lectin-like receptor subfamily G1 (KLRG1) and transcription factor T-box (T-bet), suggesting that the HA-specific CTLs acquired an effector memory (T_EM_) like phenotype (**Figures 4A, B**, **Figures S5A–C**). More than 92% of CD8^+^Thy1.1^+^ T cells displayed a naive phenotype (CD44^lo^CD62L^hi^CD127^lo^KLRG1^lo^) prior to transfer, indicating that upregulation of these markers occurred *in vivo* within the tumor-bearing animal (**Figure S5D**). Importantly, ICT responders displayed increased frequencies of CD44^hi^CD62L^lo^CD127^hi^KLRG1^hi^ T_EM_ CD8^+^Thy1.1^+^ T cells compared to non-responders in both DLNs (**Figure 4C**; P = 0.0002) and tumors (**Figure 4D**; P < 0.0001). Endogenous CD8^+^Thy1.1^−^ T cells retained a naïve phenotype (CD44^lo^CD62L^hi^) in DLNs and an effector phenotype (T_EFF_; CD44^hi^CD62L^lo^CD127^lo^KLRG1^lo^) in tumors (**Figures 4A, B**). Endogenous CD8^+^Thy1.1^−^ T cells were similar between responding and non-responding tumors and DLNs (**Figures 4C, D**), suggesting that differences in T_EM_ frequencies were mostly found in the HA-specific CTLs.

A small proportion of non-responding mice exhibited tumor infiltration of CD8^+^Thy1.1^+^ T cells (>10%). To determine whether HA-specific CTLs acquired a T_EM_ surface phenotype in these non-responding animals, we examined the expression profiles of CD127 and KLRG1 based on their median fluorescence intensity (MFI). Responders had a significantly higher CD127 MFI compared to non-responders in tumors, but not DLNs (**Figures 4E, F**; DLN: P = 0.05; Tum: P = 0.02). KLRG1 MFI was similar between responders and non-responders for both compartments (**Figures 4E, F**; DLN: P = 0.77; Tum: P = 0.08).

We questioned whether CTL function, as measured by the release of cytotoxic effector molecules and the expression of proliferation and activation markers, was associated with response to ICT. Although CD8^+^Thy1.1^+^ T cells had increased expression of Granzyme B, Ki67 and PD-1 compared to their endogenous CD8^+^Thy1.1^−^ counterparts, the frequencies for both endogenous and HA‑specific CD8^+^ T cells that expressed these markers were similar between responders and non-responders (**Figure S6**). Bulk RNAseq of CD8^+^Thy1.1^+^ TILs supported this, as we found minimal differences in immune-related gene sets between responders and non-responders, with the exception of genes associated with WNT/β-catenin signaling being upregulated in non-responders (**Figure S7**).

Together, these data suggest that HA-specific CTLs display an activated phenotype after ICT, and that animals with increased frequencies of T_EM_ HA-specific CTLs are more likely to respond to ICT. Although non-responding animals had significantly lower frequencies of tumor infiltrating HA-specific CTLs, they still exhibited a memory-like phenotype and retained cytotoxic function.

### ICT Responders Maintain a Tumor-Specific Memory T Cell Response

**Figure 5:**
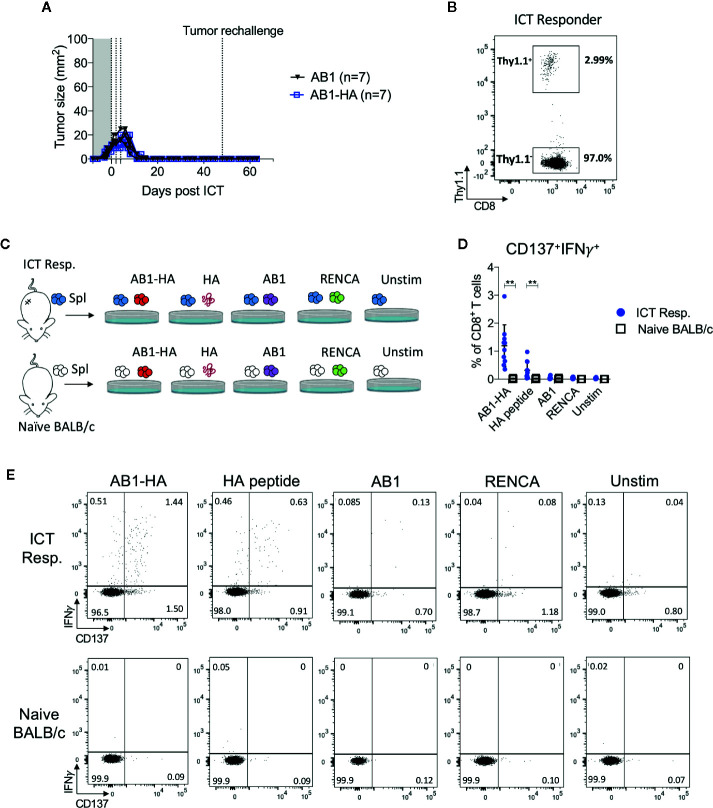
ICT responders develop a tumor antigen-specific memory T cell response. **(A)** Representative tumor growth curves of ICT responders re-challenged with AB1-HA or AB1 tumor cells 30 days after the primary tumor regressed to 0 mm^2^. **(B)** Representative FACs plot of CD8^+^Thy1.1^+^ T cells from splenocytes from an ICT responder, 15 days after AB1-HA tumor re-challenge. **(C)** *Ex vivo* co-culture setup to assess antigen-specific T cell responses. Splenocytes from ICT responders or naive BALB/c mice were co-cultured with AB1-HA, AB1, RENCA tumor cells, or HA peptide. **(D)** Dot plots and **(E)** representative flow cytometry plots, showing percentages of CD8^+^ T cells that co-expressed IFNγ and CD137 for each culture condition. Data shown as mean ± SD, summary of two independent experiments; ICT responders: n = 10; Naïve BALB/c: n = 4. Mann-Whitney *U* tests; **P ≤ 0.01.

To determine if ICT responders formed a memory T cell response against a broad range of tumor antigens, we re-challenged responding animals with AB1-HA or the parental AB1 tumor cell lines 30 days after the original tumor completely regressed. All ICT responders were protected from re-challenge of either tumor cell line (**Figure 5A**). CD8^+^Thy1.1^+^ T cells were detected in the spleen of these re-challenged animals, indicating that they persisted after tumors regressed (**Figure 5B**). CD8^+^ T cells from splenocytes of ICT responders produced IFNγ and upregulated CD137 when co-cultured with AB1-HA tumor cells (P = 0.002) and HA peptide (P = 0.007), but minimally with AB1 or MHC-I matched control (RENCA) tumor cells (**Figures 5C–E**). These results suggest that ICT responders successfully formed a long-lasting memory CD8^+^ T cell response against AB1-HA tumors.

## Discussion

Here, we studied tumor antigen (HA)-specific CTLs in ICT responders and non-responders using an established murine bilateral tumor model (22, 23, 37). We found that ICT increased tumor infiltration of HA-specific CTLs, but this increase varied between animals. Successful ICT responses were associated with increased post-treatment frequencies of effector memory (T_EM_) HA‑specific CTLs within tumors and draining lymph nodes, but not with pre-treatment frequencies. Previous murine studies demonstrated that some ICTs enhanced tumor antigen-specific CTL responses (21, 38, 39), and increased expression of activation and memory associated markers (20, 40). While these reports have added to our understanding of how ICT changes tumour antigen‑specific CTLs, the majority are limited to comparisons between ICT-treated and untreated animals. Our study adds to this as we relate ICT induced changes in tumor antigen-specific CTLs to eventual outcomes in treated mice.

Our main finding is that increased frequencies of IL-7Rα expressing HA-specific T_EM_ CTLs correlated with ICT response. The T_EM_ phenotype reported in our study is similar to memory precursor effector T cells found in chronic viral infections (41–44). IL-7/IL-7Rα signalling pathway is crucial for memory CTL formation. The effects of ICT are IL-7 dependent (45), and combination IL-7 and ICT improved therapeutic benefit and long-term memory T cell responses in murine models (46–48). Increased frequencies of IL-7Rα expressing tumor antigen-specific CTLs found in the present study could indicate increased survival and persistence of memory CTLs in the tumor microenvironment and possibly providing long-term therapeutic benefit to ICT. A recent study by Sade-Feldman and colleagues similarly found an effector/memory CTL gene signature including the IL-7R gene, that was enriched in ICT responding tumors (12). IL-7R expressing memory T cells could be a T cell population of interest that defines ICT outcomes.

A challenge lies in identifying definitive CTL populations that correlate with ICT outcomes. Memory CTLs exist in heterogeneous differentiation states expressing different combinations of surface markers CCR7, CD69, CD44, Slamf7, PD-1, and T cell factor 1 (TCF1). However, the change in different memory CTL subsets during ICT vary between studies (12, 20, 40, 49, 50). In contrast to published studies, we did not observe any difference in post-ICT PD-1 expression or proliferation (Ki67) of HA-specific CTLs between responding and non-responding animals (9, 13, 51). This highlights the complexity in memory CTL differentiation and the need to understand how these populations change during ICT, especially in relation to treatment outcome.

HA-specific CTLs present in non-responding tumors were enriched for genes involved in WNT/β-catenin signaling, which could be a mechanism underlying the difference in HA-specific CTL frequencies between responders and non-responders. Our result is supported by studies which show that increased β-catenin *in vitro* reduces CD8^+^ T cell proliferation, decreases tumor antigen-specific CTL function and promotes resistance to ICT *in vivo* (52–55). Neutralising WNT signaling *in vivo* expands tumor antigen–specific effector memory CTLs, which were phenotypically similar to our study (56). However, WNT signaling is also associated with the formation of stem-cell like memory T cells through the upregulation of TCF1, which mediate superior anti-tumor T cell responses (57). Increased proportions of CD8^+^PD-1^+^TCF1^+^ T cells associated with improved survival for ICT treated melanoma patients (11). A caveat with our study is that non-responding mice with HA-specific CTLs present in tumors are rare, and we were unable to perform an in-depth phenotypic analysis to address this discrepancy. The role of WNT/β-catenin signaling in the development of anti-tumor CD8^+^ T cell immunity requires further investigation.

In clinical studies, increased frequencies of tumor antigen-specific CTLs from pre- to post-ICT have been reported in ICT responders (5, 58). While most responding tumors generally exhibited high frequencies of HA-specific CTLs in the present study, there were some exceptions. A small proportion of animals had greater than 20% tumor infiltration of HA-specific CTLs however did not respond to ICT. This may be due to the presence of immune suppressive cells, such as myeloid derived suppressor cells and regulatory T cells, which suppressed tumor antigen-specific CTL function even if they were present (59, 60). Conversely, few animals had an absence of HA-specific CTLs but still displayed complete tumor regression following ICT. This suggested that while it is desirable to have large numbers of CTLs specific for one tumor antigen, it is not essential for response; CTLs specific for multiple tumor antigens may be required (61). The present study was limited to analysing the role of CTLs against one model tumor antigen. We did not analyse the tumor reactivity of endogenous CTLs which could have correlated to ICT response. Tracking one antigen specificity allowed us to study the effects of antigen-specific CTLs in ICT outcomes however the frequency and phenotype of multiple tumor antigen-specific CTLs in ICT should be investigated in future studies.

TCR sequencing is often used as a complementary approach to assess the breadth of antigen-specific T cell responses and is a potential biomarker of response to ICT. Successful ICT responses were associated with reduced tumor TCRβ diversity in our study, similar to other murine studies (38, 62, 63). Cancer patients with improved survival exhibit a greater expansion of TCRβ clones after ICT in their tumors and peripheral blood, compared to non-responders (16–19, 64), suggesting that effective therapy requires expansion of tumor antigen-specific CTLs. Although we were able to track the expansion of a single antigen-specific CTL clone, the dominance of this clone prevented us from studying the breadth of the anti-tumor T cell response or identifying expansion of other TCRβ clones in the endogenous CD8^+^ T cell compartment in relation to ICT outcomes. The combination of the high affinity CL4 TCR transgenic, and robust cross presentation of the HA antigen in draining lymph nodes (65, 66) could have resulted in the dominant expansion of HA-specific CTLs over other specificities in responding animals.

Apart from the introduced CL4 clone, there was minimal sharing and similarities in tumor TCRβ clones between animals. Highly private tumor TCRβ repertoires have been described in pre-clinical studies by others (38, 67) and ourselves (unpublished data). Crosby and colleagues found that ICT expanded private tumor TCRβ clones even in the presence of a fixed tumor antigen (38). This highlights the highly diverse nature of TCRβ repertoires in tumor models with limited variation. Each animal expands unique tumor TCRβ clones during ICT, and identifying cognate antigens of expanded TCR clones associated with successful ICT responses will inform personalized antigen-specific therapies.

In conclusion, post-treatment frequencies of effector memory tumor antigen-specific CTLs, and a clonal CTL repertoire correlate with response to ICT in our model. A potential dynamic biomarker of response could lie within the distribution of TCRβ clones within a memory T cell population (18). Further validation is required to investigate if this could predict ICT outcomes in cancer patients.

## Data Availability Statement

The original contributions presented in the study are included in the article/**Supplementary Material**, further inquiries can be directed to the corresponding author/s.

## Ethics Statement

The animal study was reviewed and approved by Harry Perkins Institute of Medical Research Animal Ethics Committee.

## Author Contributions

NP performed mouse experiments, analyzed and interpreted all flow cytometry, TCRβ and RNA sequencing data, and wrote the manuscript. JK performed mouse experiments and TCRβ repertoire analysis. SG performed mouse experiments. CT assisted with murine surgery experiments and RNA sequencing analysis. SF assisted generating TCRβ libraries. VF and CF performed surgery experiments. RZ and WL designed and provided technical assistance with the bilateral tumor model. AC and MW performed TCRβ and RNA sequencing. ID assisted with statistical analysis. LB provided reagents. RH provided and optimized TCRβ sequencing. RL, AN, AM, and WL interpreted experiments and critically revised the manuscript. JC designed the study, supervised the project and edited the manuscript. All authors contributed to the article and approved the submitted version.

## Funding

NP was supported by Cancer Council WA and UWA Richard Walter Gibbon Medical Research scholarships. JK was supported by an icare Dust Diseases Board scholarship. WL was supported by a Simon Lee Fellowship, an NHMRC Fellowship, and a Cancer Council WA fellowship. JC was supported by grants and fellowship from the UWA Raine Foundation, Cancer Council WA, WA Department of Health, and icare Dust Diseases Board. The National Centre for Asbestos Related Diseases receives funding through the National Health and Medical Research Council Centres of Research Excellence scheme.

## Conflict of Interest

LB was employed by the company Polpharma Biologics.

The remaining authors declare that the research was conducted in the absence of any commercial or financial relationships that could be construed as a potential conflict of interest.
