# Supplementary material for: Tumor Infiltrating Effector Memory Antigen-Specific CD8+ T Cells Predict Response to Immune Checkpoint Therapy
Source: Front Immunol. 2020 Nov 12;11:584423. doi: 10.3389/fimmu.2020.584423 (PMC7688517; doi:10.3389/fimmu.2020.584423)
Supplement: Supplementary file 1 [file DataSheet_1.docx]

Supplementary Material

**Table S1**. Flow cytometry antibodies used for T cell phenotyping and T cell sorting.

| **Antibodies for T cell panel 1** | | | | | | |
| --- | --- | --- | --- | --- | --- | --- |
| **Fluorochrome** | **Antigen** | **Dilution** | **Clone** | **Supplier** | **Catalog No.** | **RRID** |
| BUV395 | CD3 | 1:250 | 145-2C11 | BD | 563565 | AB_2738278 |
| Zombie UV | FVD | 1:4000 |  | Biolegend | 423107 |  |
| BV421 | CD44 | 1:1000 | 1M7 | Biolegend | 103040 | AB_2616903 |
| BV510 | CD8a | 1:250 | 53-6.7 | BD | 563068 | AB_2687548 |
| BV605 | Tbet | 1:500 | 4B10 | Biolegend | 644817 | AB_11219388 |
| BV711 | CD127 | 1:250 | A7R34 | Biolegend | 135035 | AB_2564577 |
| BV786 | CD4 | 1:1000 | RM4-5 | BD | 563727 | AB_2728707 |
| FITC | Foxp3 | 1:500 | FJK-16s | eBioscience | 11-5773-82 | AB_465243 |
| PE | Thy1.1 (CD90/90.1) | 1:500 | 12.14 | eBioscience | 202524 |  |
| PE-Cy7 | KLRG1 | 1:1000 | 2F1/KLRG1 | Biolegend | 138415 | AB_2561735 |
| APC | CD45 | 1:500 | 30-F11 | Biolegend | 103112 | AB_312977 |
| AF700 | CD62L | 1:1000 | MEL-14 | BD | 560517 | AB_1645210 |
| **Antibodies for T cell panels 2-4 & T cell sorting** | | | | | | |
| V500 | Live (ef506) | 1:1000 |  | eBioscience | 65-0866-14 |  |
| PerCP-Cy5.5 | CD4 | 1:500 | GK1.5 | Biolegend | 100434 | AB_893324 |
| PE-Cy7 | CD3 | 1:500 | 17A2 | Biolegend | 100220 | AB_1732057 |
| APC | CD45 | 1:500 | 30-F11 | Biolegend | 103112 | AB_312977 |
| APC-eFlour780 | CD8a | 1:500 | 53-6.7 | eBioscience | 47-0081-82 | AB_1272185 |
| PE | Thy1.1 (CD90/90.1) | 1:500 | 12.14 | eBioscience | 202524 |  |
| Pacific Blue | Gran B | 1:250 | GB11 | Biolegend | 515407 | AB_2563195 |
| BV421 | PD-1 | 1:250 | 29F.1A12 | Biolegend | 135217 | AB_10900085 |
| AF488 | Ki67 | 1:250 | 11F6 | Biolegend | 151204 | AB_2566800 |
| PE | IFN𝛾 | 1:250 | XMG1-2 | eBioscience | 12-7311-82 | AB_466193 |
| APC | CD137 | 1:500 | 17B5 | eBioscience | 17-1371-82 | AB_2573162 |

**Figure S1. Adoptive transfer of CL4xThy1.1 splenocytes did not affect ICT response rate.** BALB/c mice were transferred with either CL4xThy1.1 (CL4 spl) or BALB/c (BALB/c spl) splenocytes one day before single flank s.c. AB1-HA inoculation. When tumors reached 9-20 mm^2^, mice were treated with either ICT (anti-CTLA-4 and anti-PDL1) or PBS. Tumor growth was monitored. No significant difference in survival was found between ICT treated groups that were transferred with BALB/c or CL4xThy1.1 splenocytes. P > 0.05. Mantel-Cox survival test.

**Figure S2. Absolute numbers of HA-specific CD8^+^ T cells are increased in ICT responding tumors 7 days post ICT. (A)** Dot plots comparing; tumor sizes, frequencies of CD45^+^ cells and total cell numbers of DLN and tumors between responders (R; blue) and non-responders (NR; red) at day 0 (top) and day 7 (bottom). Graphs showing T cell subsets **(B)** as a frequency of CD45^+^ cells and **(C)** absolute numbers in DLN (top) and tumors (bottom) at both time points for responders and non‑responders. Data shown as mean ± SD, summary of two independent experiments. Mann-Whitney *U* tests; *P ≤ 0.05, **P ≤ 0.01, ***P ≤ 0.001, ****P≤ 0 .0001.

**Figure S3. Frequency of intra-tumoral T_regs_ significantly correlates with frequency of HA‑specific CD8^+^ T cells post ICT treatment**. Linear regression analysis between CD4^+^Foxp3^+^ T cells and CD8^+^Thy1.1^+^ T cells in DLN and tumors at day 0 **(A)** and 7 **(B).**

**Figure S4. ICT responders have a more clonal TCRβ repertoire than non-responders. (A)** The total number of TCRβ sequences significantly correlated with the number of sorted CD8^+^ T cells. **(B)** Dot plots representing that the number of sorted CD8^+^ T cells, total TCRβ sequences and unique TCRβ clones are similar between responders (R) and non-responders (NR). **(C)** Non-responders have a more diverse TCRβ repertoire compared to responders as shown with Shannon’s entropy (P = 0.019). **(E)** Shannon’s entropy significantly correlated with the frequency of CL4 TCRβ clones.

**Figure S5. Tumor antigen-specific CD8^+^ T cells have increased expression of activation and memory markers compared to endogenous CD8^+^ T cells post ICT. (A)** Representative FACs plots gating CD8^+^Thy1.1^+^ (blue) and CD8^+^Thy1.1^-^ (grey) T cells in DLN and tumors for CD44^hi^CD62L^lo^ and CD44^lo^CD62L^hi^ phenotypes. **(B)** Dot plots representing frequencies of CD44^hi^CD62L^lo^ and CD44^lo^CD62L^hi^ of both CD8^+^ T cell subsets between responders (R) and non-responders (NR). **(C)** Histograms showing increased expression of CD127, KLRG1 and T-bet on CD44^hi^CD62L^lo^ CD8^+^Thy1.1^+^ T cells. **(D)** Representative FACs plots showing CD44, CD62L, CD127 and KLRG1 expression on CD8^+^Thy1.1^+^ T cells from naïve CL4xThy1.1 splenocytes prior to adoptive transfer into BALB/c recipients.

**Figure S6. Expression of activation, proliferation and cytotoxic markers on CTLs are similar between ICT responders and non-responders. (A)** Representative histograms and adjacent dot plots of CD8^+^Thy1.1^+^ (blue) and CD8^+^Thy1.1^-^ T cells (grey), expressing PD-1, Ki-67 and granzyme B (GrB) in post ICT draining lymph nodes (DLN) and tumors (Tum). FMO control for each marker are represented as a black dotted line on each histogram plot. **(B)** Graphs comparing frequencies of CD8^+^Thy1.1^+^ and CD8^+^Thy1.1^-^ T cells expressing PD-1, Ki-67 and GrB between responding (R) and non-responding (NR) DLNs and Tum. Data on graphs represented as mean ± SD, summary of two independent experiments (R: n = 11; NR: n = 5). Mann-Whitney *U* tests were used to compare between both responders and non-responders, and between CD8^+^Thy1.1^+^ and CD8^+^Thy1.1^-^ T cells for each phenotypic marker; *P ≤ 0.05, **P ≤ 0.01, ***P ≤ 0.001, ****P ≤ 0.0001.

**Figure S7. Tumor antigen-specific CD8^+^ T cells in non-responding tumors are enriched with genes associated with WNT/β-Catenin signaling**. GSEA displaying top hallmark gene sets in CD8^+^Thy1.1^+^ T cells in responding (NES > 0) vs non-responding (NES < 0) tumors. WNT/β-Catenin Signaling and Protein Secretion gene sets are significantly (q < 0.25) enriched in CD8^+^Thy1.1^+^ T cells in non-responding tumors. n = 3 biological replicates. NES: Normalized Enrichment Score

**Figure S8. Gating strategies for FACS analysis. (A)** Gating strategy for T cell panel 1 (Table 1) to analyze expression of CD44, CD62L, CD127, KLRG1 and T-bet on CD8^+^Thy1.1^+^ and Thy1.1^-^ T cells. CD127 (orange) and KLRG1 (red) FMOs are overlayed on CD44^hi^CD62L^lo^ CD8^+^ T cell subsets. **(B)** Gating strategy for T cell panels 2, 3 and 4 (Table 1) to analyze expression of PD-1, Ki-67 and Granzyme B on CD8^+^Thy1.1^+^ and Thy1.1^-^ T cells, and CD137 and IFN𝛾 on CD8^+^ T cells.

**Figure S9. Gating** **strategy for sorting CD8 T cells from post-ICT treated tumors.** Total CD8^+^ T cells for TCRβseq analysis and CD8+Thy1.1^+^ T cells for RNAseq analysis were sorted using the BD Melody with antibodies outlined in table 1. Red boxes define sorted population gates.
